# Efficacy of integrated school based de-worming and prompt malaria treatment on helminths -*Plasmodium falciparum *co-infections: A 33 months follow up study

**DOI:** 10.1186/1472-698X-11-9

**Published:** 2011-06-22

**Authors:** Nicholas Midzi, Sekesai Mtapuri-Zinyowera, Davison Sangweme, Noah H Paul, Godfrey Makware, Munyaradzi P Mapingure, Kimberly C Brouwer, James Mudzori, Gibson Hlerema, Vivian Chadukura, Francisca Mutapi, Nirbhay Kumar, Takafira Mduluza

**Affiliations:** 1 National Institute of Health Research, Box CY 573, Causeway Harare, Zimbabwe; 2 College of Health Sciences, Department of Medical Microbiology, P.0 Box A178, Avondale, Harare, Zimbabwe; 3 Johns Hopkins Bloomberg School of Public Health, Department of Molecular Microbiology and Immunology, Baltimore, Maryland, USA; 4 University of Zimbabwe, Department of Biochemistry, P.O Box MP167, Mount Pleasant, Harare, Zimbabwe; 5 Central Statistical Office, PO Box CY 342, Causeway, Harare, Zimbabwe; 6 University of California, San Diego, Division of International Health & Cross Cultural Medicine, Department of Family and Preventive Medicine, San Diego, California USA; 7 National Microbiology Reference Laboratory, P.O. Box ST749, Southerton, Zimbabwe; 8 University of Edinburgh, Institute for Immunology and Infection Research, Edinburgh, UK

**Keywords:** co-infection, malaria, schistosomiasis, STHs, deworming

## Abstract

**Background:**

The geographical congruency in distribution of helminths and *Plasmodium falciparum *makes polyparasitism a common phenomenon in Sub Saharan Africa. The devastating effects of helminths-*Plasmodium *co-infections on primary school health have raised global interest for integrated control. However little is known on the feasibility, timing and efficacy of integrated helminths-*Plasmodium *control strategies. A study was conducted in Zimbabwe to evaluate the efficacy of repeated combined school based antihelminthic and prompt malaria treatment.

**Methods:**

A cohort of primary schoolchildren (5-17 years) received combined Praziquantel, albendazole treatment at baseline, and again during 6, 12 and 33 months follow up surveys and sustained prompt malaria treatment. Sustained prompt malaria treatment was carried out throughout the study period. Children's infection status with helminths, *Plasmodium *and helminths-*Plasmodium *co-infections was determined by parasitological examinations at baseline and at each treatment point. The prevalence of *S. haematobium, S. mansoni*, STH, malaria, helminths-*Plasmodium *co-infections and helminths infection intensities before and after treatment were analysed.

**Results:**

Longitudinal data showed that two rounds of combined Praziquantel and albendazole treatment for schistosomiasis and STHs at 6 monthly intervals and sustained prompt malaria treatment significantly reduced the overall prevalence of *S. haematobium, S. mansoni*, hookworms and *P. falciparum *infection in primary schoolchildren by 73.5%, 70.8%, 67.3% and 58.8% respectively (p < 0.001, p < 0.001, p < 0.001, p < 0.001 respectively). More importantly, the prevalence of STH + schistosomes, *P. f *+ schistosomes, and *P. f *+ STHs + schistosomes co-infections were reduced by 68.0%, 84.2%, and 90.7%, respectively. The absence of anti-helminthic treatment between the 12 mth and 33 mth follow-up surveys resulted in the sharp increase in STHs + schistosomes co-infection from 3.3% at 12 months follow up survey to 10.7%, slightly more than the baseline level (10.3%) while other co-infection combinations remained significantly low. The overall prevalence of heavy *S. haematobium*, *S. mansoni *and hookworms infection intensities were significantly reduced from: 17.9-22.4% to 2.6-5.1%, 1.6-3.3% to 0.0% and 0.0-0.7% to 0.0% respectively.

**Conclusion:**

Biannual Integrated school based antihelminthic and sustained prompt malaria treatment has a potential to reduce the burden of helminths-*plasmodium *co-infections in primary school children. In areas of stable malaria transmission, active case finding is recommended to track and treat asymptomatic malaria cases as these may sustain transmission in the community.

## Background

Millions of people in Sub Saharan Africa are infected with neglected tropical diseases (NTDs) that include schistosomiasis (200 million), hookworms (198 million), *Ascaris lumbricoides *(173 million) and *Trichuris trichiura *(182 million) among others [1-3 ]. Evidence also suggests that the same continent is overwhelmed with a burden of malaria. In 2009, World Health Organisation indicated that of the global 243 million cases of malaria reported in 2008, 208 million cases were from Africa alone [4].

*S. haematobium *and *S. mansoni *are schistosomes of medical importance in Zimbabwe. *S. haematobium *is transmitted over greatest area in Zimbabwe. Several studies have shown the prevalence of schistosomiasis ranging from 52-80% [5-8]. Schistosomiasis is rated among the top 10 causes of hospital outpatient attendance in Zimbabwe [9]. Although some studies reported occurrence of soil transmitted helminthiasis (STH) in some parts of the country with hookworm prevalence ranging from 26%- 88% [8,10-12], there is no data describing the extent of distribution of STHs in Zimbabwe. Soil transmitted helminthiasis does not appear in the National annual health profile reports [9].

Malaria is widely distributed in Zimbabwe. *Plasmodium falciparum *accounts for 97.8% of malaria species of medical importance in the country. This is followed by *P. malariae *and *P. ovale *with prevalence of 1.8% and 0.3% respectively [13]. Malaria occurs all year round in some districts and annually top 20 districts are selected as high transmission areas. Districts like Mutasa, Chimanimani and Mutare located in the eastern highlands always appear among the annual top 20 districts with high incidence of malaria [9]. In 2008 alone malaria was responsible for the national hospital inpatient cases of 11 350 and the national inpatient death of 232 [9]. The number of primary school aged children diagnosed with clinical malaria in the year 2008 in Zimbabwe was 210 994 [9].

The geographical congruence of socio economic and climatic conditions that favour overlap in spatial distribution of schistosomes, (STHs) and *Plasmodium falciparum *(poverty, poor hygiene status, lack of protective clothing, lack of clean water and poor sanitation, as well as limited access to preventive measures and health care), made helminths-*Plasmodium falciparum *co-infection a common phenomenon in Sub Saharan Africa [8,14-16]. It has been observed that helminths--*Plasmodium falciparum *co-infection exacerbates clinical out comes of malaria and its frequency of attacks [17-20] although some studies show a protective effect [[21,22], and [23]].

In total, 45.1 million (25%) school-aged children in Sub Saharan Africa are at coincidental risk of hookworm and malaria infection [14]. Thus the most intense infections with the commonest worms occur in school-age children with low protective immunity against malaria [24]. Single helminths, *Plasmodium falciparum *and helminths -*Plasmodium falciparum *co-infections are known to cause anaemia [4,25-28] and reduced school attendance [29,30]. They also impair childhood growth [31], intellectual development, reduce school performance [[32,33], and [34]] and reduce worker productivity [35]. Schistosomiasis and STHs are responsible for

over 415 000 annual deaths and 43.5 million DALYS [16]. Hence, malaria and the NTDs are of huge public health and economic significance [4,36] and their successful control is a key issue towards progress of the millennium development goals [16].

Owing to the burden of morbidity imposed by helminth-*Plasmodium falciparum *co-infections in primary schoolchildren and the proven geographical congruency in the distribution of helminths and *Plasmodium falciparum *in the developing world [14,37], the global interest for integrated control of NTDs has increased. Hotez et al [16] suggested incorporating a rapid - impact package for NTDs with programmes for HIV/AIDS, tuberculosis and malaria (the big three diseases) that have attracted major funding support for control. The World Health Organisation advocates cost effective preventive chemotherapy, and encourages all its member states to integrate NTDs control intervention strategies in order to control co-morbidity due to co-infections [38]. On the other hand provision of intermittent preventive treatment (IPT), long lasting insecticide treated nets (LLINs) in schools, health education for prevention and prompt malaria treatment combined with regular treatment with albendazole and praziquantel have been suggested [37,39,40]. However as there is no empirical data on the impact of implementing different mixes of interventions for control of helminths -*Plasmodium falciparum *co-infections, rigorous community based efficacy and feasibility studies have been recommended for any integrated control strategy in order to inform on the best practices that will improve the quality of life in primary schools [16,37-41].

A study was therefore conducted to evaluate the parasitological efficacy of integrated school based antihelminthic praziquantel and albendazole treatment as well as prompt malaria treatment on the prevalence of schistosomiasis, STHs, malaria and helminths -*Plasmodium falciparum *co-infections in rural and commercial farming areas in Zimbabwe. The effect of treatment intervals on prevalence of polyparasitism and on helminths infection intensities was also assessed.

## Methods

### Study design, area and population

A school based longitudinal intervention study that involved examination and treatment of the study population for malaria, schistosomiasis and STHs at baseline, and again during 6, 12 and 33 months follow up surveys was conducted among primary school age children (5-17 years). Sustained prompt malaria treatment was carried out throughout the study period." The Baseline survey was conducted in June to July 2004. Follow up studies were conducted in November to December 2004, June 2005 and March 2007 respectively. The study population comprised of children living in Nyamaropa rural area located in Shamva district, Mashonaland Central province and Burma Valley commercial farming area located in Mutare district, Manicaland province. The study areas are described in detail elsewhere [28]. Inhabitants in Nyamaropa rural area use the perennial Mupfurudzi River and Eben dam as water sources used for market gardening, laundry, fishing and bathing. Water sources in Burma Valley commercial farming area include perennial rivers flowing from the range of mountains in the east and dams that provide constant supply of water for irrigation and recreation. Soils are constantly wet and maintain water ponds as a result of irrigation activities practiced in large banana plantations in the farming area. Generally the climate of Zimbabwe is tropical. There is a dry season, including a short cold season during the period May to August when the whole country has very little rain. The dry hot season occurs during the period of September to early November, while the rainy season is typically a time of heavy rainfall from November to March, when the Inter-tropical Convergence Zone influences the whole country. This is rainfall experienced in Nyamaropa and Burma Valley commercial farming areas. However due to the existence of a range of high mountains in the eastern highlands of Zimbabwe where Burma Valley is located, relief rainfall is experienced during the rainy season. Thus the commercial area receives high annual rainfalls.

The sample size was obtained using n = (z/Δ)^2 ^p(1-p), where n is the sample size required, z = 1.96 is the z-score associated with a 95% confidence interval (CI), Δ is the margin of error = 0.05 and p is the prevalence of disease. Based on previous studies in Mutare District, the prevalence of hookworm, 61.7% [12], S. haematobium, 58.7% [12] and malaria, 23.5% [42] were used. The prevalence of *S. haematobium *(53.1%) observed in Chiweshe and Bushu areas adjacent to Nyamaropa in Shamva district, Mashonaland Central [43] was used to calculate the sample size in the rural area. The largest of the four sample sizes (n = 373) was considered optimal and it was adjusted by 30% to n = 485 considering possible loss due to follow up.

Multistage sampling technique was used to select districts, wards and schools in which the study was conducted. Using rotary method, Mutare district was randomly selected out of the 7 districts in Manicaland province. Ward 7 was randomly selected from the Mutare district out of which 3 primary schools were also selected. Shamva district was randomly selected out of 7 districts of Mashonaland Central province and ward 10 was selected from 29 wards in the district. Nyamaropa Primary School, with an enrolment of over 700 pupils and the only school in Ward 10 was included in the study. Every child from Grade 1 to 6 attending each of the selected schools was eligible for the study. Excluded from the study were Grade 7 children since they could not be followed up for two years as they would have transferred to secondary schools. Also excluded were severely sick children, those who could not provide stool, urine or blood samples and those not willing to participate in the study.

All demographic data were obtained by interviewing children. Age of also obtained from the class registers provided by teachers when available.

#### Parasitological techniques

Urine and faecal samples were collected between 10:00 am and 2:00 pm in separate wide mouth plastic specimen bottles correspondingly labelled with the laboratory identification numbers assigned to each individual. The samples were processed within two hours of collection. Diagnosis of *S. haematobium *and intestinal helminths (*S. mansoni*, hookworms, *T. trichiura *and *A. lumbricoides*) was based on the detection of worm eggs in urine and faeces, respectively. The urine filtration technique as described by Mott *et al *[44] was used for diagnosis of urinary schistosomiasis. Urine samples were collected from each individual and microscopically examined on three successive days in order to prevent misdiagnosis due to day-to-day variation of egg excretion [45].

In order to improve sensitivity in diagnosis of intestinal helminths (*S. mansoni*, hookworm, *T. trichiura, A. lumbricoides*), two methods, Kato Katz and the formal ether concentration technique, were used. Results from the two techniques were combined to determine intestinal helminths infection status. In addition results from the two Kato Katz slides prepared on two successive days (one slide/day) were used to determine egg intensities for intestinal helminths. On day one of urine collection, a stool sample was collected from each participant as well. The stool was processed using the formal ether concentration technique as described by Cheesbrough [46] and examined for the presence of hook, *T. trichiura, A. lumbricoides *and *S. mansoni *ova. Stools samples collected from each individual on day 2 and 3 were processed using the Koto Katz technique as described by Katz *et al *[47]. On each day one slide of a thick smear was prepared from each specimen and examined for STHs ova within one hour of preparation in order to visualise and quantify hookworm ova before they degenerated. Thick smears were left to clear for 24 hours after which they were re-examined for *S. mansoni *infection. In determining the infection status of intestinal helminths, a person who had ova in stool as observed using either the formal ether concentration technique, Kato Katz technique or had ova detected in stool using both techniques was considered positive for the parasite species demonstrated by the ova observed. The individual was considered negative if no ova were detected in stool using both techniques. The number of ova observed for each intestinal helminths using the Kato Katz technique was multiplied by 24 in order to get the number of eggs per gram stool (epg). Since two slides were examined on two successive days the mean egg intensity was calculated by determining the mean of the sum of ova detected on day 2 and day 3 (two days).

Approximately 5 ml of venous blood was drawn from willing participants in blood collection tubes containing ethylene diamine tetra acetic acid (EDTA) as anticoagulant. Thick smears of malaria slides were prepared from fresh blood and the remaining blood was used for plasma separation being used for immunological studies elsewhere. *P. falciparum *was diagnosed by microscopic examination of thick blood films after staining the smears with Giemsa stain [46]. The presence of either ring forms or gametocytes was conclusive diagnosis of *P. falciparum *infection after reading fields through 200 white blood cells.

### Interventions

#### (a) Basic life skills education in schools

The research team provided basic health education about malaria; schistosomiasis and STHs in schools. Children were taught about malaria risks and were asked to continuously seek prompt malaria treatment based on recognition of signs and symptoms of the disease that include fever, headache, nausea, general malaise and joint pains. Leaflets with basic information about malaria, STHs and schistosomiasis where distributed to children in schools involved in the study for children to learn basic life skills to avoid diseases under investigation. At each school, teachers were taught about the life cycles of malaria, schistosomiasis, STHs and signs and symptoms of these diseases so that they would teach their children regularly during their free or study periods. A flip chart with basic information about bilharzia was given to the school headmaster who was responsible for circulating it in turns to each class from grade 1 to 7. Class teachers were asked to continue school health education on schistosomiasis, STHs and malaria and to encourage children to seek medical care promptly when they felt signs and symptom of malaria.

#### (b) Treatment

Children infected with any of the schistosome species and STHs were treated with Praziquantel at 40 mg/kg body and a single albendazole tablet 400 mg as a single dose by the state registered nurse. Bread and orange juice (500 ml/child) were given as supplementary food following swallowing of tablets in order to reduce the nauseating effect of Praziquantel and to aid absorption. Combined deworming refers to treatment of schistosomiasis and STHs at the same treatment time point: baseline, 6, 12 and 33 months follow up surveys. Treatment for malaria was based on parasitological results from the microscopic examination of Giemsa stained blood thick smears at baseline, and also during 6, 12 and 33 moths follow up surveys when the research team was in the field. All children who had either gametocytes or ring forms in their blood thick smears were taken to the nearest clinic for treatment. During the time when the research team was not in the field participants also sought prompt malaria treatment based on their ability to recognise signs and symptoms of the disease as taught in school. Malaria was treated with a combination of chloroquine, sulphadoxine and pyrimethamine (SP) according to local malaria case management guidelines [48].

## Prompt malaria treatment monitoring

A register was left with the nurse in charge of Burma Valley clinic who also served as our research nurse during the field visits. All children reporting for malaria treatment in the absence of the research team were recorded in the register. The research team collected this information on subsequent follow up surveys. Also children were asked if they had received malaria treatment during the period in between the surveys.

### Ethical consideration

The Medical Research Council of Zimbabwe gave ethical approval for the study. In addition, Provincial and District Medical and Education Directors, chiefs, councillors and village head-men granted permission. General information regarding the nature of study and objectives was explained to the community and study participants. Feedback and consent was sought at schools, farms and village meetings. Inclusion of children into the study took place after free individual, parental and school authority informed consent. Children joined the study voluntarily and were allowed to drop out at any time they wished without any prejudice.

#### Data management and analysis

Data was captured using SPSS version 8. Frequency tables together with 95%CI were obtained. The percentage reduction of the prevalence of parasites from baseline to 33 months follow up survey and geometric mean egg counts was calculated. χ^2^- test was used where appropriate. McNemar's test was used to test for significant difference in prevalence of parasites between baseline and 33 months follow up surveys Helminths infection intensities were classified according to the World Health Organization guidelines [1]. *S. haematobium *infection was classified into light infection (1-49e/10 ml urine) and heavy infection (≥ 50e/10 ml urine). *S. mansoni *infection determined using the Kato Katz technique was stratified into light (1-99epg stool), moderate (100-399epg stool), heavy infection (≥ 400epg stool). Hookworm infection intensity determined using the Kato Katz technique was stratified into light infection (1-1999epg stool), moderate infection (2000 - 3999epg stool) and heavy infection (≥ 4000epg stool). Single infection referred to infection with schistosomes (*S. haematobium *and or *S. mansoni*), any one of or all of the STHs or *P. falciparum*. Co-infection referred to infection with schistosome + STH, *P. falciparum *+ schistosomes, *P. falciparum *+ STHs or *P. falciparum *+ STHs + schistosomes.

In order to assess the prevalence and effect of integrated school based deworming and prompt malaria treatment on helminths - *Plasmodium *co-infections, parasites infection combinations were stratified at each treatment point into the following groups: (0) non infected individuals, (1) schistosomiasis infection only, (2) STHs infection only, (3) *P. falciparum *infection only, (4) infection with schistosomes + STHs, (5) infection with *P. falciparum *+ schistosomes, (6) infection with *P. falciparum *+ STHs, (7) infection with *P. falciparum *+ STHs + schistosome.

## Results

Table 1 describes the baseline demographic data for study participants. Overall 1303 participants were recruited into the study from both sites (Nyamaropa rural area and Burma Valley commercial farming areas). The overall mean age (standard deviation) of the study population was 10.1 (2.6) years. The mean ages (standard deviation) of study participants in Burma Valley farming and Nyamaropa rural area were 10.3 (2.3) and 9.9 (2.2) years respectively. Overall, there were more males (52%) than female (52%). The helminthic disease with the highest prevalence in both rural and commercial farming areas was schistosomiasis. Malaria and soil transmitted helminthiasis were only observed in Burma Valley commercial farming area.

**Table 1:** Baseline comparison of parasitic distribution between rural area and farming area in Zimbabwe.

Variable	Overall	Burma Valley farming area	Nyamaropa rural area	**χ**^**2**^
*P. falciparum*				
Number examined	935	512 (100)	423 (100)	
Infected n (%)	143 (15.3)	143 (27.9)	0	0.001
*S. haematobium*				
Number examined n (%)	1279	599 (100)	680 (100)	
Infected n (%)	767 (60.0%)	313 (52.3)	454 (66.8)	0.001
*S. mansoni*				
Number examined	1249	577 (100)	672 (100)	
Infected n (%)	214 (17.10	131 (22.7)	83 (12.4)	0.001
*Hookworms*				
Number examined	1249	575 (100)	674 (100)	
Infected n (%)	137 (11.0)	136 (23.7)	1 (0.1)	0.001
*A. lumbricoides*				
Number examined	1249	575 (100)	674 (100)	
Infected n (%)	12 (1.0)	12 (2.1)	0	0.001
*T. trichiura*				
Number examined	1249	575 (100)	674 (100)	
Infected n (%)	13 (1.0)	13 (2.3)	0	0.001

### Effect of integrated school deworming and prompt malaria treatment on the prevalence of schistosomes, STHs ad P. falciparum

The effect of integrated school based deworming and prompt malaria treatment on the prevalence of schistosomes, STHs and *P. falciparum *was determined from those children who were successfully followed up at all surveys and had all parasitological results (n = 682).

The effect of intervention on the prevalence of *P. falciparum*, schistosomes and STHs in Burma Valley farming area and on schistosomiasis only in Nyamaropa rural area at each follow up time point is shown in table 2 and 3.

**Table 2:** Effect of intervention on prevalence of schistosomiasis among 262 primary school children in Nyamaropa rural area in Zimbabwe

Parasite	Prevalence (95%CI)			
	Baseline	6 months	12 months	33 months
***S. haematobium***				
**Overall**	65.3(59.2-71.0)	27.5(22.2-33.3)	18.3(13.8-23.5)	34.0 (28.3-40.1)
**Sex**				
Males	70.9(62.1-78.6)	27.6(20.0-36.2)	18.9(12.5-26.8)	37.0(28.6-46.0)
Females	60.0(51.2-60.3)	27.4(20.1-35.7)	17.8(11.7-25.3)	31.1(23.4-39.6)
**S. mansoni**				
**Overall**	9.5(6.3-13.8)	7.3(4.4-11.1)	5.7(3.3-9.3)	0.0
**Sex**				
Males	7.1(3.3-13.0)	6.3(2.8-12.0)	7.9(3.8-14.0)	0.0
Females	11.9(6.9-18.5)	8.1(4.1-14.1)	3.7(1.2-8.4)	0.0

**Table 3:** Effect of intervention on prevalence of *P. falciparum *and hookworms at different time points among primary schoolchildren in Burma Valley farming area (n = 420) in Zimbabwe

Parasite	Prevalence (95%CI)			
	Baseline	6 months	12 months	33 months
***P. falciparum***				
**Overall**	28.3(24.1-32.9)	12.9(9.8-16.4)	12.4(9.4-15.9)	8.1(5.7-11.1)
**Sex**				
Males	26.1(20.4-32.5)	11.5(7.6-16.5)	12.4(8.3-17.5)	9.2(5.7-13.8)
Females	30.7(24.4-37.6)	14.4(9.8-20.0)	12.4(8.2-17.7)	6.9(3.8-11.4)
***S. haematobium***				
**Overall**	55.2(50.3-60.1)	14.3(11.1-18.0)	13.3(10.2-17.0)	30.7 (26.3-35.4)
**Sex**				
Males	52.8 (45.9-59.5)	12.4 (8.3-17.5)	13.3 (9.1-18.5)	34.4 (18.1-41.1)
Females	57.9 (50.8-64.8)	16.3 (11.5-22.2)	13.4 (9.0-18.4)	26.7 (20.8-33.4)
***S. mansoni***				
**Overall**	22.6(18.7-26.9)	4.0(2.4-6.4)	4.5(2.7-7.0)	17.4(13.9-21.4)
**Sex**				
Males	28.4 (22.6-34.9)	3.2 (1.3-6.5)	4.6 (2.2-8.3)	15.6 (11.1-21.1)
Females	16.3 (11.5-22.2)	5.0 (2.4-8.9)	4.5 (2.1-8.3)	19.3 (14.1-25.4
**Hookworm**				
**Overall**	23.8(19.8-28.2)	8.6(6.1-11.7)	7.1(4.9-10.0)	15.7(12.4-19.6)
**Sex**				
**Males**	27.5(21.7-34.0)	9.6(6.1-14.3)	7.3(4.3-11.6)	15.6(11.0-21.1)
**Females**	19.8(14.5-26.0)	7.4(4.2-12.0)	6.9(3.8-11.4)	15.8(11.1-21.6)

There was a significant decrease in prevalence of *P. falciparum *from baseline to 33 months follow up survey (28.3% to 8.1%) with the percentage prevalence reduction of 54.4%, 56.2% and 71.9% from baseline to 6, 12 and 33 months follow up surveys respectively. McNemar's test demonstrated a significant reduction in prevalence of malaria from baseline to 33 months follow up survey, p < 0.001. There was no significant difference in the prevalence of malaria between males and females at each treatment point in time. Throughout the study period, malaria was not diagnosed parasitologically in the rural area.

Following the first two rounds of treatment at six monthly intervals during the first year, there was a steep decline in prevalence of *S. haematobium *in both rural and commercial farming areas. The observed overall percentage reduction (PR) of *S. haematobium *was 75.9% in the commercial farming area and 72.0% in the rural area during the first 12 months of intervention. The absence of anti-helminthic treatment between the 12 mth and 33 month follow-up surveys resulted in rebounding of *S. haematobium *prevalence in both areas although it did not reach the baseline level (tables 2 and 3). In the commercial farming area, the prevalence of *S. haematobium *infection was not different between males and females at each follow up survey. In the rural area, the baseline prevalence of *S. haematobium *was significantly higher among males than females but there was no significant difference in prevalence at 6, 12 and 33 months follow up surveys (table 3).

**Figure 1:**
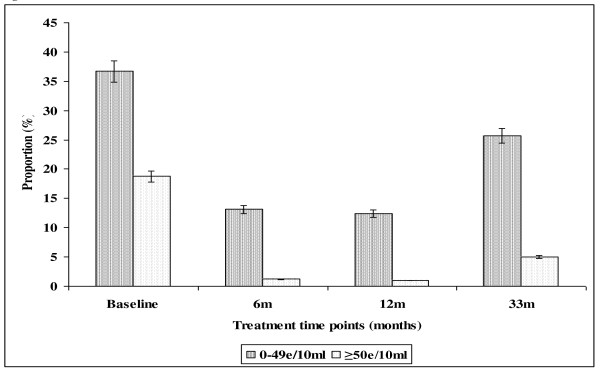
**Effect of integrated school based praziquantel and albendazole treatment for schistosomiasis and STHs on proportion of *S. haematobium *infection intensities among primary schoolchildren living in Burma Valley farming area in Zimbabwe**.

In the farming area, the prevalence of light *S. haematobium *infection intensity declined progressively from baseline (36.7%) to 12 months (12.4%), PR = 66.2%, after which it increased to 25.7% at 33 months follow up survey (Figure 1). The overall prevalence of heavy infection intensity significantly declined from 18.8% (95%CI = 15.3-22.9) at baseline through 1.2% (95%CI = 0.4-2.8) at 6 months follow up treatment point to 1.0% (95%CI = 0.3-2.4) at 12 months follow up survey. This corresponded to the percentage reduction of 94.7% after 2 rounds of treatment. The absence of anti-helminthic treatment between the 12 months and 33 months follow-up surveys resulted in a slight increase in the prevalence of heavy infection intensity to 5.0% (95%CI = 3.1-7.5) (Figure 1).

**Figure 2:**
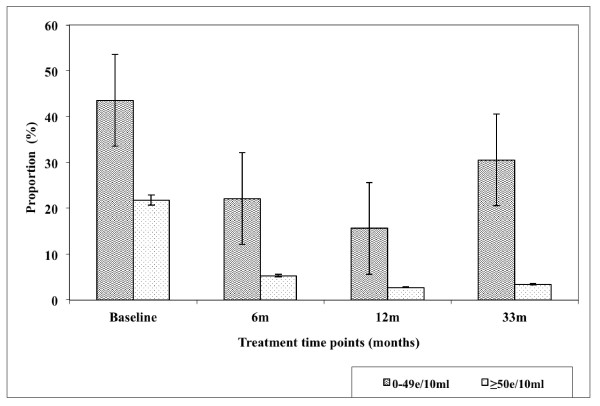
**Effect of integrated school based praziquantel and albendazole treatment for schistosomiasis and STHs on proportion of *S. haematobium *infection intensities among primary schoolchildren living in Nyamaropa rural area in Zimbabwe**.

Figure 2. Demonstrate the effect of intervention on the prevalence of both light and heavy infection with *S. haematobium *over 33 months in Nyamaropa rural area. The prevalence of light infection declined progressively from 43.5% (95%CI = 37.4-49.8) at base line to 15.6% (95%CI = 11.5-20.6) at 12 months follow up survey. It rebounded to 30.5% (95%CI = 25.0-36.5) as a result of the absence of treatment between 12 months and 33 months follow up surveys. Heavy infection declined progressively from baseline 21.8% (95%CI = 16.9-27.2) to 2.7% (95%CI = 1.1-5.4) at 12 months follow up survey after which it slightly increased to 3.4% (95%CI = 1.6-6.4) at 33 months follow up survey.

**Figure 3:**
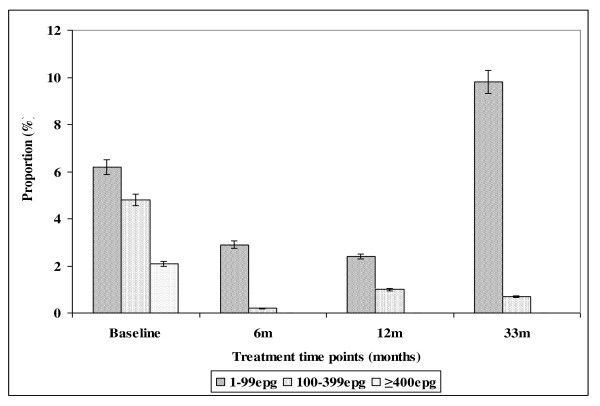
**Effect of integrated school based praziquantel and albendazole treatment for schistosomiasis and STHs on the proportion of *S. mansoni *infection intensities among primary schoolchildren living in Burma Valley farming area in Zimbabwe**.

**Figure 4:**
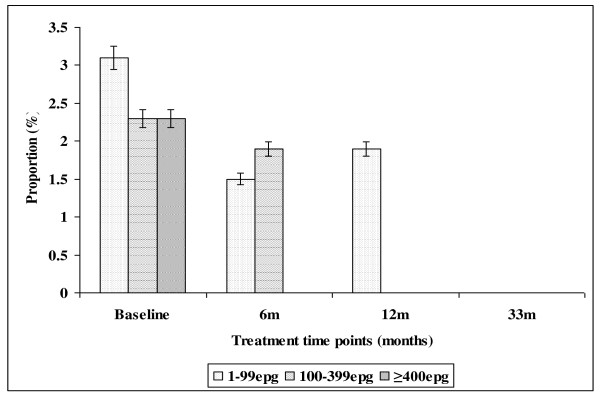
**Effect of integrated school based praziquantel and albendazole treatment for schistosomiasis and STHs on the proportion of *S. mansoni *infection intensities among primary schoolchildren living in Nyamaropa rural area in Zimbabwe**.

The effect of intervention on the prevalence of *S. mansoni *and its infection intensities in both Burma Valley farming area and Nyamaropa rural area is illustrated in tables 2 and 3, Figure 3 and Figure 4 respectively. The prevalence of light infection showed a stead decline from 6.2% (95%CI = 4.1-8.9) at baseline to 2.4% (95%CI = 1.1-4.3) at 12 months follow up survey in Burma Valley farming area. The absence of treatment between 12 months and 33 months follow up surveys resulted in the rebounding of prevalence exceeding the base line level to 9.8% (95%CI = 7.1 - 13.0). Heavy infection was 2.1% (95%CI = 1.0-4.0) at baseline and this was cleared with the first round of treatment (Figure 3). In Nyamaropa rural area, the prevalence of light infection, 3.1% (95%CI = 1.3-5.9) at baseline was cleared with the first three rounds of treatment. Moderate infection intensity, 4.8% (95%CI = 2.9-7.3) at baseline was cleared with the first two rounds of treatment. The first round of treatment at baseline cleared heavy infection (Figure 4).

The overall prevalence of hookworm in Burma Valley farming area was 23.8% (95%CI = 19.8-28.2) at baseline (Table 2). Following two rounds of treatment and school based health education, the overall prevalence of hookworm infection in the farming area declined to 5.1% at 12 months follow up survey then increased to 15.7% at 33 months follow up survey. McNemar's test showed a significant decline in proportion of children infected with hookworms from baseline to 33 months follow up survey, p = 0.004. Males had a higher prevalence of hookworm infection than females at base line although this was not significant (χ^2 ^= 3.4, p = 0.063).

**Figure 5:**
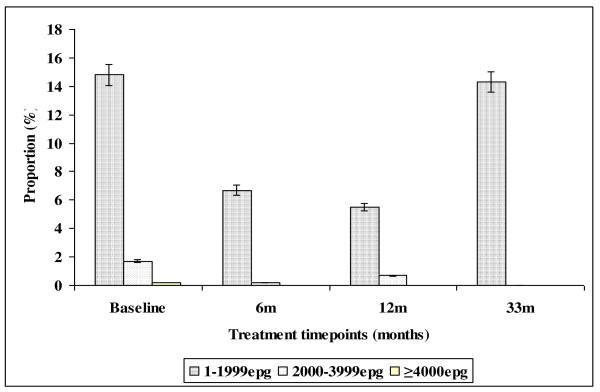
**Effect of integrated school based praziquantel and albendazole treatment for schistosomiasis and STHs on the prevalence of hookworm infection intensities among primary schoolchildren living in Burma Valley farming area in Zimbabwe**.

The effect of intervention on hookworm infection intensity over 33 months follow up period is illustrated in Figure 5. The prevalence of light infection intensity declined from 14.8% (95%CI = 11.5-18.5) at baseline to 5.5% (95%CI = 3.5-8.1) following the first two rounds of treatment in the first year (PR = 64.1%). However at 33 months follow up survey, the prevalence of hookworm light infection shoot up to 14.3% (95%CI = 11.1-18.0). Moderate hookworm infection intensity was cleared with the first three rounds of treatment in the first year.

### Effect of integrated school based deworming and prompt malaria treatment on prevalence of helminths - Plasmodium falciparum co-infections

The impact of integrated treatment strategy on co-infections is described in table 4. The proportion of children who had no infection after all parasites had been screened was 21.4%. This increased to 70.0% following two rounds of treatment in the first year. The absence of treatment between 12 and 33 months resulted in the proportion declining to 49.0% at 33 months follow up survey. Prevalence reduction of children infected with schistosomiasis only was 65.3% following two rounds of treatment in the first year. The prevalence of children infected with STHs decreased from 5.7% at baseline to 3.6% following two rounds of treatment in the first year and that of children infected with *P. falciparum *increased to 9.0 at 12 months follow up survey.

**Table 4:** Effect of integrated school based de-worming and prompt malaria treatment on helminths -*Plasmodium *co-infections among 420 primary schoolchildren successfully followed up over 33 months in Burma Valley farming area, Zimbabwe

Co-infection combinations	Prevalence (95%CI)				
	Baseline	6 months	12 months	33 months	Overall (%) reduction
Not infected	21.4 (17.6-26.7)	65.2 (60.5-69.8)	70.0 (65.4-74.3)	49.0 (44.2-53.9)	-
Schistosomiasis	31.7 (27.2-36.4)	5.5 (3.5-8.1)	11.0 (8.1-14.3)	27.6 (23.4-32.2)	3.9
STHs	5.7 (3.7-8.4)	5.5 (3.5-8.1)	3.6 (2.0-5.8)	4.5 (2.7-7.0)	6.3
*P. f*	8.1 (5.7-11.1)	10.5 (7.7-13.8)	9.0 (6.5-12.2)	4.0 (2.4-6.4)	40.9
Schisto + STHs	12.9 (9.8-16.4)	3.6 (2.0-5.8)	3.8 (2.2-6.1)	10.7 (7.9-14.1)	17.1
*P. f *+ schisto	13.1 (10.0-16.7)	1.2 (0.4-2.8)	1.9 (0.8-3.7)	2.6 (1.3-4.6)	73.7
*P. f *+ STHs	1.4 (0.5-3.1)	1.0 (0.3-2.4)	0.2 (0.0-1.3)	0.5 (0.1-1.7)	63.6
*P. f *+ STHs + schisto	5.7 (3.7-8.4)	0.2 (0.0-1.3)	0.5 (0.0-1.7)	1.0 (0.3-2.4)	76.4

The rural area was left out in this analysis because there were no mixed infections observed over the period of the study.

Key: Schisto = schistosomiasis, *P. f *= *P. falciparum*, STHs = soil transmitted helminths

The prevalence percentage reduction of co-infection with schistosomes + STHs was 70.5% after two rounds of treatment in the first year. However, the proceeding 21 months of delayed treatment resulted in an exceptional increase in prevalence of schistosome + STHs co-infection (10.7%). Following two rounds of treatment in the first year the percentage prevalence reduction of *P. falciparum *+ schistosome was 85.5%. However this declined to 80.2% at 33 months follow up survey. The PR for co-infection with *P. falciparum *+ STHs was 85.7% at 12 months follow up survey. Co-infection with *P. falciparum *+ STHs + schistosomiasis declined from 5.7% at baseline to 0.5% following two rounds of treatment in the first year (PR = 91.2%). However the PR declined to 82.5% with delayed treatment at 33 months follow up survey.

Overall the prevalence of children infected with at least two parasites at baseline, 6, 12 and 33 months follow up surveys in the commercial farming area were 33.1% (1139/420), 5.5% (23/420), 6.4% (27/420) and 15.0% (63/420) respectively.

### Monitoring of prompt malaria treatment

Following baseline treatment and subsequent school health education, the following participants reported to the nearest health facility (Burma valley clinic) between baseline and six months follow up survey (n = 8), between 6 months and 12 months follow up survey (n = 24) and between 12 months and 33 months follow up survey (n = 56).

## Discussion

Our study investigated efficacy of combined school based antihelminthic treatment with Praziquantel and albendazole; prompt malaria treatment and health education in control of schistosomiasis, STHs, malaria and on suppression of helminths - *Plasmodium *co-infections. Results from our study demonstrate that two rounds of deworming and sustained prompt malaria treatment significantly reduce the proportions of children with helminths -*Plasmodium falciparum *co-infections. The observed low level of helminths-*Plasmodium falciparum *co-infection during the proceeding 21 months (almost 2 years) could have been due to the initial combined treatment intervention and school based health education leading to change of children's behaviour towards the preventive practices. Thus the intervention could be promising as it managed to reduce the proportion of helminths-*Plasmodium falciparum *co-infection, hence the multiplicative effect of co-infection on malaria clinical outcomes and morbidity [17,20,27,28,49,50].

If similar but large scale intervention could demonstrate similar results to our study then application of such a strategy in helminths -*Plasmodium falciparum *co-endemic areas would result in reduction of morbidity associated with helminths *Plasmodium falciparum *co-infections or helminths co-infections [29,30,32-34]. Successful implementation of such a strategy at national level in endemic countries could be a cost effective way of delivering drugs for treatment of multiple diseases affecting primary school children. Reduction of morbidity due to polyparasitism resulting from combined school based drug administration and prompt malaria treatment could contribute towards achievement of the Millennium Development Goals number 2 and 6: Universal primary education and disease eradication, respectively [16,36,40]. The pragmatic target set by the World Health Organisation: to regularly treat at least 75% of primary school children at risk of morbidity due to schistosomiasis and STHs by the year 2010 [51] could also be achieved. However the set target could be achieved later than 2010 since until only a few of the endemic countries has mapped areas with overlapping NTDs or helminths - *Plasmodium falciparum *co-infections for control [16,37,41,52].

The observed higher prevalence of co-infection with schistosomes + STHs in Burma Valley farming area following 21 months of delayed treatment than that observed at pre-treatment survey as well as the unprecedented increase in prevalence of *S. mansoni *and hookworm light infections (figures 3 and 5) in Burma Valley farming area, are of concern. However they could have little impact on morbidity and hence school health since these are all helminths whose effect is dependent on infection intensity rather than prevalence [1,3,41]. The observed low levels of heavy infection intensities (Figures 1, 2, 3, 4 and 5) that is critical for morbidity due to helminthiasis [1,3,30] even when re-treatment was delayed by almost 2 years demonstrate that regular biannual de-worming could still be efficacious with regards to morbidity control especially in areas of low STHs infection intensities as observed Burma Valley farming area, a strategy also shown to decrease S. haematobium infection in Burkina Faso [53]. This treatment interval could make national helminthiasis control programmes feasible in the developing world whose resources are limited.

Currently, conflicting suggestions on the timing of regular deworming do exist, with the World Health Organization recommending regular annual de-worming in areas where the prevalence of schistosomiasis is ≥ 50% and the prevalence of STHs ≥ 20%, whilst the best practice paper on deworming considers it cost effective if annual deworming is done when the prevalence of STHs is ≥ 40% [38,41]. Empirical results from our study show that for a prevalence of 23.8% observed in the farming area at baseline (Table 2), the proportion of individuals with heavy infection was less than 1% (Figure 5). These results agree with Hall and Horton's calculated 0.01 proportions of individuals at risk of high STHs infection intensities when the prevalence is 20% [41,51]. Thus the threshold prevalence of ≥ 20%, recommended by WHO for annual STHs de-worming could be none cost effective if Hall and Horton's economic calculations on treatment rounds based on prevalence are to be considered [38].

The prevalence of STHs > 50% was observed in school children attending Kinyasini and Chaani approximately 6 months after a decade of sustained regular school based de-worming [54]. This was attributed to local risk factors that included socio-economic discrepancies, poor hygienic practices and soil composition [54]. Many of the children who participated in our study were from unemployed subsistence farmers in the rural area or the lowly paid farm worker communities whose farming activities involved regular irrigation of banana plantations and tobacco, thus the soils in the farming communities are always moist enough to support survival of STHs eggs and larvae hence transmission. These risk factors associated with transmission of schistosomiasis and STHs [41] could have caused the exceptional resurgence of helminths light infections and schistosome + STHs co-infections observed in our study after almost two years of delayed treatment (Figures 1, 2, 3, 4 and 5).

The observed significant difference in prevalence of *S. mansoni *in Nyamaropa and Burma Valley commercial farming areas at 33 months follow up survey demonstrate the effect of heterogeneities in water contact patterns in different communities and the spatial heterogeneity in the distribution of infected snails on the epidemiology of schistosomiasis [55-57]. In areas where infected intermediate host snail population is limited as could be the case for Nyamaropa rural area, the transmission force of the disease is low and repeated treatment can reduce the disease to a level where its public health significance would be no more. However areas like Burma Valley farming area where intermediate host snails could be significantly populated and water reservoirs are perennial (personal observation), water contact activities will continue all year round resulting in resurgence of the disease if treatment is delayed by two years (Table 2). However only light infection rebounded after almost 2 years of delayed treatment in our study (Figure 3,), making the decision to de-worm biannually optimal and cost effective in similar endemic areas in the developing world where resources are already strained.

Although the research team and school teachers educated children about malaria disease, its signs and symptoms, a strategy implemented continuously through out the study to promote children to recognise malaria and seek prompt treatment, Table 2 shows that still a certain percentage of children could be found with *Plasmodium falciparum *parasites at each treatment point parasitologically. These could be a few asymptomatic malaria cases whose immunity could be able to control parasite load and tolerate parasitaemia without clinical manifestation of the disease [58]. Thus in stable malaria endemic areas like Burma Valley farming area, additional active case finding is recommended to track and treat asymptomatic malaria cases as these may sustain transmission of the disease in the community although this has an implication on cost-effectiveness of the approach. Provision of LLINs and their effective use would complement the effort as IPT in schools [59,60] could be irrational today given the cost of the new malaria drug compounds and the need to prevent rapid emergence of resistance resulting from drug abuse.

The health seeking behaviour of children for malaria treatment as demonstrated by records made at the clinic is difficult to attribute it to school health education intervention since the figures of children who reported for prompt malaria treatment were rather low at each follow up survey. There was also no control arm. We observed that at each follow up survey, some children harboured *P. falciparum *parasites but did not seek treatment. The three primary schools in Burma Valley are served with Burma Valley clinic that is located at the centre and is approximately 200 m away from Msapa primary school, and about 2 to 3 km away from Kaswa and Valhalla primary schools. The schools are therefore in close proximity to the clinic, thus distance can not be attributed to low compliance observed. More studies in diverse endemic communities are recommended in order to explore the role of school based health education on prompt malaria treatment. Although our study show that integrated school based parasite control can reach a wide population at risk of infection and resulted in reduction of prevalence of polyparasitism, teachers were not able to provide treatment since the local policy stipulates that only medically qualified personnel prescribe and administer drugs regardless of safety guaranteed. In order to realise maximum benefit from school based integrated treatment approach, school teachers need to be allowed to administer treatment in situations where drugs are declared safe like praziquantel and albendazole as this is not only cost effective but also a quick strategy for reaching children living in hard to reach areas who are most commonly overburdened with curable neglected tropical disease.

To our knowledge, this is the first study to investigate the efficacy of combined school based control strategy for helminths - *Plasmodium *co-infections. Whilst the results show some potential, it should be noted that the study did not have an untreated control arm due to ethical reasons, a weakness that begs for care in interpreting our results. Further, rigorous studies are therefore recommended in diverse areas in order to inform public health managers on appropriate and cost effective integrated control measures in areas co-endemic for NTDs and malaria.

## Conclusion

Biannual Integrated school based anthelminthic and sustained prompt malaria treatment has a potential to reduce the burden of helminths-*plasmodium *co-infections in primary school children. In areas of stable malaria transmission, active case finding is recommended to track and treat asymptomatic malaria cases as these may sustain transmission in the community.

## Competing interests

The authors declare that they have no competing interests.

## Authors' contributions

TM, NM, NK, KCB and FM contributed to the concept and design of the study protocol; NM, DS, SZ, TM, NHP and VC carried out the clinical assessment and parasitology; TM, NM, MG, and MPM carried out the analysis and interpretation of the data; NM and TM drafted the manuscript. All authors read and approved the final manuscript. NM and TM are guarantors of the paper.

## Funding

The UNICEF/UNDP/World Bank/WHO Special Programme for Research and Training in Tropical Diseases Grant A60125, ENHR-Ministry of Health and child Welfare, Harare, Zimbabwe, Fogarty Grant for field work to NK and DS. International Foundation for Science Grantee: W/4321-1 to TM.

## Pre-publication history

The pre-publication history for this paper can be accessed here:

http://www.biomedcentral.com/1472-698X/11/9/prepub
